# Comparative study of stenting and ostium packing in Endoscopic Dacryocystorhinostomy for Primary Acquired Nasolacrimal Duct Obstruction

**DOI:** 10.1038/s41598-019-57019-0

**Published:** 2020-01-08

**Authors:** Joyce Chin, Vincent Lam, Regine Chan, C. L. Li, Luke Yeung, Antony Law, Alvin Young, Hunter Yuen, Mohammad Javed Ali, Kelvin K. L. Chong

**Affiliations:** 1 Department of Ophthalmology and Visual Sciences, Prince of Wales Hospital, 30-32 Ngan Shing Street, Shatin, New Territories Hong Kong; 2 Department of Ophthalmology and Visual Sciences, The Chinese University of Hong Kong, 4/F, Hong Kong Eye Hospital, 147K Argyle Street, Kowloon, Hong Kong; 3 Hong Kong Eye Hospital, 147K Argyle Street, Kowloon, Hong Kong; 4 L.V. Prasad Eye Institute, Kallam Anji Reddy Campus, L. V. Prasad Marg, Banjara Hills, Opp. PVR, Hyderabad, Telangana 500034 India; 5 Present Address: Hahnemann University Hospital, 230 North Broad Street, Philadelphia, PA 19102 United States

**Keywords:** Lacrimal apparatus diseases, Surgery

## Abstract

In this retrospective study, we compared the efficacy and safety of mechanical adjuvants in mucosal-sparing, mechanical endoscopic dacryocystorhinostomy (MMED) for primary acquired nasolacrimal duct obstruction (PANDO). 116 adult patients (90 female) aged 61 ± 11 received one of the following after MMED without topical mitomycin: no stenting or packing (group 1, n = 25), 1-week ostium packing by ribbon gauze (group 2, n = 29) or non-medicated absorbable gelatin sponge (group 3, n = 25), 8-week bicanalicular stenting (group 4, n = 28). 104 patients(92%) provided 12-month outcomes. Number of patients, age, gender, surgeon, and osteotomy size were comparable among groups (p = 0.4–0.9). Marginal significance was found in anatomical (group 1:80%, group 2:96.6%, group 3:96%, group 4:96.4%, p = 0.05) but not functional success (group 1:85%, group 2:85.7%, group 3:83.3%, group 4:88.9%, p = 0.75) at postoperative 12-month. Patients receiving any packing or stenting achieved better anatomical (96% versus 80%, p = 0.015) but not functional success (85% versus 86%, p = 0.90) compared to those receiving none. More patients receiving stenting developed postoperative granuloma than those who did not (87% versus 63%, p = 0.04). 1-week ostium packing was found to be as effective as 8-week bicanalicular intubation in improving anatomical outcome after MMED for PANDO. Functional outcome, however, did not differ among patients receiving mechanical adjuvant or not.

## Introduction

Primary acquired nasolacrimal duct obstruction (PANDO) was defined as complete resistance to lacrimal irrigation with 100% regurgitation from the same or opposite punctum, or the presence of a lacrimal sac mucocele without secondary causes^1^. Endonasal dacryocystorhinostomy (DCR) was proposed since 1893 by Cadweli^2^, with varieties of surgical techniques described including laser endoscopic DCR, endonasal endoscopic mechanical dacryocystorhinostomy, mucosal-preserving mechanical endoscopic dacryocystorhinostomy (MMED)^3^. Synechial adhesion with middle turbinate and/or nasal septum, and progressive cicatricial closure related to secondary intention of healing with/without granuloma formation are two prevalent causes of DCR failure regardless of the surgical approaches^4^. Different operative adjuvants were described for endonasal endoscopic mechanical DCR including bicanalicular stenting^5^, intraoperative or postoperative topical mitomycin C^4^, and postoperative packing by absorbable or non-absorbable materials with or without medications e.g. topical steroid. In a prior randomized clinical trial (SEND), we showed no significant difference in postoperative 12-month outcome whether stenting was used or not after MMED, along with 1-week non-absorbable ostium packing for PANDO^6^. In this study, we compared the use of 8-week stenting, 1-week packing with control after MMED for PANDO.

## Methods

This was a retrospective comparative case series. Medical records of consecutive patients aged ≥18 who underwent mucosal-sparing, mechanical endoscopic dacryocystorhinostomy (MMED) without topical mitomycin C from December 2010 to February 2014 at the Prince of Wales Hospital, Hong Kong, were reviewed. This study was conducted according to the Declaration of Helsinki, with approval from The Joint Chinese University of Hong Kong – New Territories East Cluster Clinical Research Ethics Committee. The need for informed consent of study was waived by the local ethics committee as this was a retrospective review. All patients referred to our lacrimal clinic first underwent standardized examination of tear film, anterior segments, eyelids, and puncta. The presence and level of lacrimal obstruction were then assessed by lacrimal irrigation and probing. Endoscopy was performed in each patient before surgical scheduling for significant nasal pathologies including deviated nasal septum or active rhinosinusitis. All patients provided informed consent for surgery before operation. In this study, we excluded patients with acute dacryocystitis, canalicular obstruction, punctal stenosis, suspected lacrimal sac malignancy, facial paralysis, eyelid laxity or malposition and those with history of lacrimal operation, head and neck irradiation or trauma, reflex tearing due to ocular surface diseases or diseases affecting the ipsilateral nose and orbit.

Standard MMED with opposing mucosal flaps was performed under general or local anesthesia, according to patient preference by two experienced oculoplastic surgeons KC and CL^6^. After mucosal flaps were raised, “Cold steel” method were used to create osteotomy with 2 mm Kerrison rongeurs. Osteotomy was set superiorly at least 2 mm above the internal punctum down to the sac-duct junction inferiorly. Anterior-superiorly, the orbicularis (Horner’s) muscle was often exposed, and the agger nasi cells or the operculum of the middle turbinate was frequently entered posterosuperiorly for exposure of the lacrimal sac fundus. The lacrimal sac was then incised and marsupialized with anterior and posterior flaps with a crescent blade^6^. No topical mitomycin C was used. 11 patients had middle turbinoplasties and 1 patient had posterior bony septoplasty performed by the operating lacrimal surgeon to improve middle meatal access. After the SEND study^6^, which we showed no difference in 12-month outcome between stenting and packing or packing alone, there was no institutional protocol as to whether any mechanical adjuvant including stenting and/or packing be used routinely in PANDO. After MMED, patients received one of the following according to surgeon’s preference: no stenting or packing (group 1), 1-week ribbon gauze ostium packing (group 2), 1-week non-medicated, absorbable gelatin sponge ostium packing (group 3), or 8-week bicanalicular stenting (group 4). We prescribed eye-drops and nasal spray up to postoperative 4 weeks and prophylactic oral antibiotics were given for 1 week when non-absorbable packing was *in-situ*. Patients were followed up at week 1, 2, 4, 8, 12, 16, 26 and 52. Office endoscopies and photographs were performed at each visit. Findings were recorded on a standard datasheet used in our prior study^6^. In this study, the primary outcome was efficacy based on anatomical and functional success. Anatomical success was defined as patency on lacrimal irrigation with or without a positive endoscopic fluorescein dye test (FEDT) result^7^. FEDT is positive when topical fluorescein applied to the inferior conjunctival cul-de-sac spontaneously flows from the rhinostomy site viewed by a nasal endoscope within 20 seconds^8^. Functional success is defined as a Munk’s score (Table 1)^9^ of ≤1 in patients with anatomical success^10^.

**Table 1:** Assessment of tearing by Munk’s Score.

Grade	Clinical finding
0	No epiphora
1	Occasional epiphora requiring dabbing less than twice a day
2	Epiphora requiring dabbing 2–4 times per day
3	Epiphora requiring dabbing 5–10 times per day
4	Epiphora requiring dabbing more than 10 times per day
5	Constant tearing

**Figure 1:**
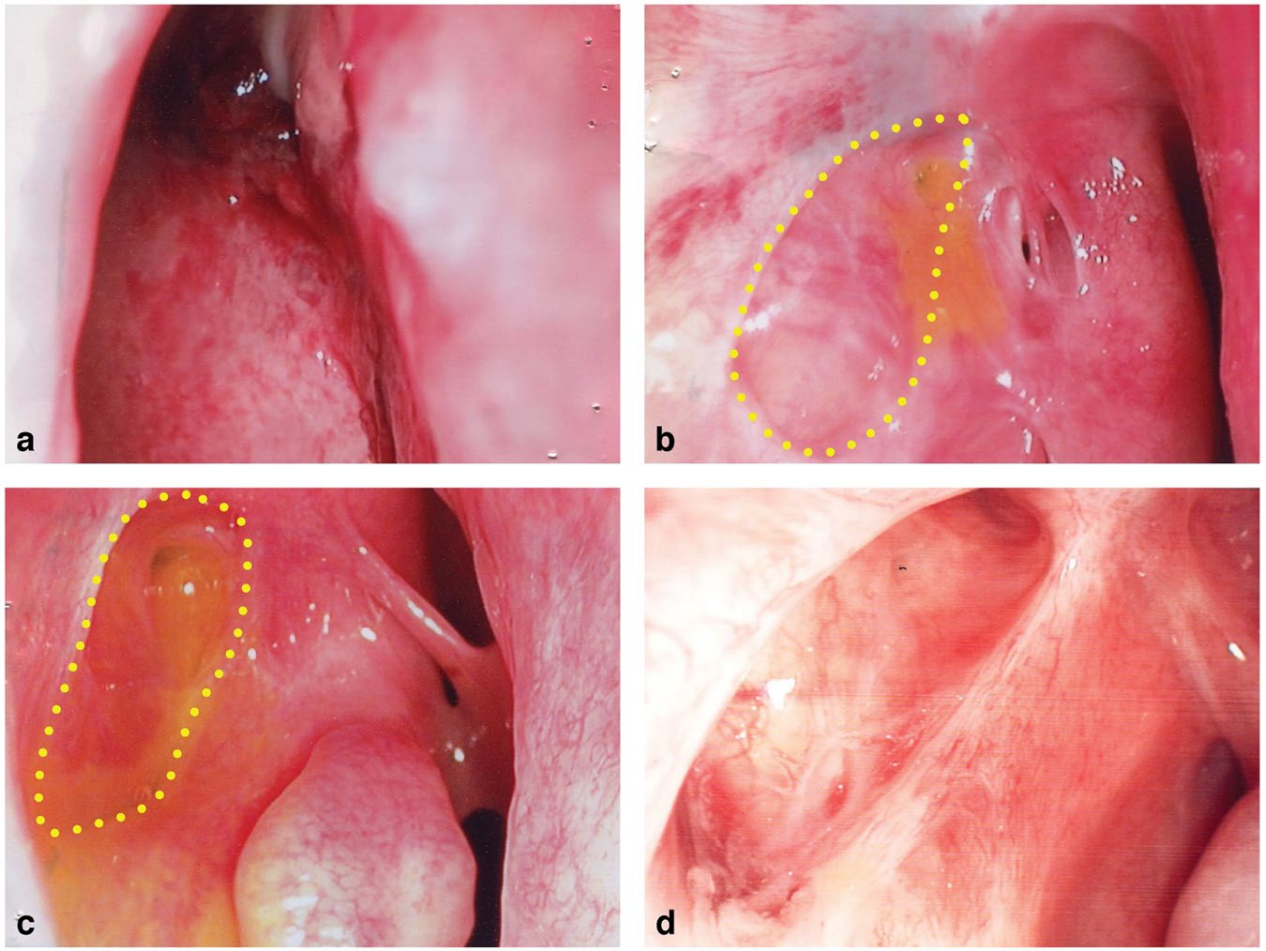
Representative endoscopic pictures showing grading of ostium shape after Mucosal-Sparing, Mechanical Endoscopic Dacryocystorhinostomy (MMED). Grade 1 (**a**) Not recognizable. Grade 2 (**b**) Flat ostium without clear border between lacrimal sac and nasal mucosa. Grade 3 (**c**) Depressed ostium with clear border between lacrimal sac and nasal mucosa. Grade 4 (**d**) Deep, wide-open ostium with clear border between lacrimal sac and nasal mucosa.

**Figure 2:**
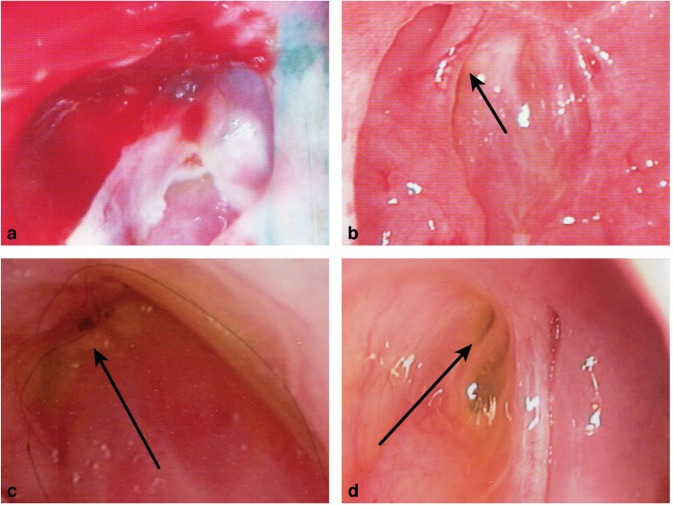
Representative endoscopic pictures showing classification of internal canalicular opening (ICO) after MMED. Grade 1 (**a**) Not recognizable. Grade 2 (**b**) Partially obstructed. Grade 3 (**c**) Overhanging mucosa. Grade 4 (**d**) Uncovered opening.

Secondary outcomes included endoscopic photos of the nasal ostium, internal canalicular opening (ICO) and surgery-related complications. Our grading system for ostium shape and ICO was based on that proposed by Ali *et al*. in 2014. The DCR ostium is defined as the postoperative opening containing the common or individual canaliculus and the surrounding (lacrimal sac) mucosal lining located in the lateral nasal wall with anterior, posterior, superior, and inferior edges in the parasagittal plane. The ICO is the junction between the distal end of common or individual canaliculus into the lacrimal sac. Representative examples of ostium shape and ICO used in our grading system were shown in Figs. 1 and 2. Three independent, trained observers masked on the outcome and grouping graded the ostium shape and ICO based on the endoscopic photos. A biostatistician arranged re-grading until two or all graders reached consensus. Surgery-related complications were retrieved from medical records up to postoperative week 52.

Statistical analyses were performed using STATA (version 14, StataCorp, College Station, TX). Continuous data were reported as mean ± SD, and Kruskal Wallis test was used to evaluate the differences among groups. Categorical data were expressed in frequencies and percentages, and Fisher’s exact test was used. Multiple comparisons were further conducted with nominal p values reported. Logistic regression was used to perform univariate and multivariate analyses for the binary outcomes. Bilateral correlation was adjusted by the mixed effect model. p values < 0.05 were considered statistically significant.

## Results

After applying the exclusion criteria, 113 adult patients underwent MMED without anti-metabolite for uncomplicated PANDO from 2010 December to 2014 February. 104 (92%) attended follow-up at postoperative 52 weeks. Results of 107 cases (3 patients received bilateral MMED) from 104 patients were available for analysis. Mean age of patients was 61.3 ± 11.3 (range: 29–86), 90 were female. 25 cases were in group 1 (no stenting or packing), 29 in group 2 (1-week ribbon gauze packing at ostium), 25 in group 3 (1-week absorbable gelatin sponge packing at ostium), and 28 in group 4 (8-week bicanalicular stenting). The four groups were comparable in age, gender ratio, laterality, anesthesia, operating surgeons, and osteotomy size (all p > 0.40) (Table 2).

**Table 2:** Baseline characteristics (fellow eye included, n = 107).

Group	1	2	3	4	p value
Adjuvant	None	Gauze packing	Gelfoam packing	Silicone stenting	n.a.
Number of cases	25	29	25	28	n.a.
Age, (mean ± SD, range)	61.4 ± 10.6 (46–83)	61.7 ± 11.5 (35–86)	61.7 ± 13.0 (35–86)	60.3 ± 10.8 (42–86)	0.40
Female (%)	22 (88.0)	24 (82.8)	21 (84.0)	23 (82.1)	0.97
Local anesthesia (%)	17 (68.0)	19 (64.5)	14 (56.0)	16 (57.1)	0.76
Surgeon A (%)	11 (44.0)	13 (44.8)	9 (36.0)	11 (39.3)	0.92
Osteotomy (mm)	20.6 ± 3.9	20.7 ± 1.92	20.15 ± 3.20	20.14 ± 4.40	0.90

Ninety-nine (92.5%) of the 107 cases achieved anatomical success at week 52. Among the 8 failed cases, 5 were in group 1 and 1 in each group 2, 3 and 4. Mean time to failure was 19.7 ± 17.7 (range: 2–52) weeks. Six were found to have progressive cicatrical ostial closure, one developed membranous obstruction (group 3) while one had small ostium not patent on irrigation (group 4). Except this last patient with small ostium who received balloon dacryoplasty, intubation and mitomycin C, 6 of the other 7 patients underwent revision MMED with stenting and mitomycin C^11^. All 7 patients who received secondary interventions achieved anatomical success at postoperative 52 weeks. Five out of these 8 patients developed granuloma after primary MMED at postoperative 6.4 ± 6.02 (range: 2–16) weeks. One was peri-tubal, one was “bang-on” and the remaining two were diffuse granulomas^12^. All were ostial-threatening and debrided during office endoscopies.

Anatomical success rates were 80.0%, 96.6%, 96.0%, 96.4% in group 1, 2, 3 and 4 respectively, reaching statistical significance (p = 0.05) (Table 3). Odds ratio (OR) of anatomical success for group 2, 3 and 4 were 7.0 (95% CI: 0.76–64.61), 6.0 (95% CI: 0.65–55.66), 6.75 (95% CI: 0.73–62.37) when compared to group 1 (OR = 1). In subgroup analysis comparing group 1 (no stenting or packing) versus group 2, 3 and 4 (either stenting or packing), patients receiving any stenting or packing had statistically significant higher anatomical success rate (96.3%) compared to those without (80.0%, p = 0.015). There was no difference among patients in group 2, 3 or 4 (all p values > 0.1). No significant difference was found between anatomically successful and failed cases in terms of age (p = 0.89), gender (p = 0.47), laterality (p = 0.93), mode of anesthesia (p = 0.96), operating surgeon (p = 0.60), osteotomy size (p = 0.41), granuloma (p = 0.89) or adjuvant received (p = 0.053). Only ICO (p = 0.026) but not ostium shape grading (p = 0.10) was significant after multivariate analysis for anatomical success.

**Table 3:** Postoperative anatomical and functional outcomes at 12 months (fellow eye included, n = 107).

Group	1	2	3	4	p value
Adjuvant	None	Gauze packing	Gelfoam packing	Silicone stenting	n.a.
Number of cases	25	29	25	28	n.a.
Anatomical failure (%)	5 (20.0)	1 (3.4)	1 (4.0)	1 (3.6)	0.05*
Functional failure (%)	3 (15.0)	4 (14.3)	4 (16.7)	3 (11.1)	0.75
Time^a^ (weeks)	21.7 ± 18.6	30.0	36.0	8.0	0.78
Granuloma (%)	14 (63.6)	16 (61.5)	15 (65.2)	20 (87.0)	0.24
Time^b^ (weeks)	6.13 ± 4.81	8.82 ± 3.36	10.3 ± 10.3	7.05 ± 4.57	0.26
Ostium grade (1:2:3:4) (%)	3:8:8:6 (12:32:32:24)	0:10:6:13 (0:35:21:45)	0:6:7:12 (0:24:28:48)	0:4:5:19 (0:14:18:68)	0.003*
ICO grade (1:2:3:4) (%)	3:4:3:15 (12:16:12:60)	1:3:7:10 (4:10:24:62)	0:3:7:15 (0:12:28:60)	0:1:9:18 (0:4:32:64)	0.10

^a^Time to anatomical failure. ^b^Time to first appearance of granulation tissue.

14 out of the 99 cases with anatomical success developed functional failure (Munk’s score >1 despite patency on irrigation and positive FEDT) at week 52. 3 patients were in group 1, 4 in group 2, 4 in group 3, 3 in group 4. No significant difference was found in functional success among intervention groups at postoperative 12 months (group 1:85.0%, group 2:85.7%, group 3:83.3%, group 4:88.9%, p = 0.75) (Table 3). During subgroup analysis, there was no difference in functional success comparing group 1(85.0%) versus group 2, 3 and 4(86.1%) (p = 0.90). There was no difference between functionally successful (n = 85) and failed cases (n = 14) in terms of gender (p = 0.48), laterality (p = 0.48), anesthesia (p = 0.07), operating surgeons (p = 0.43), osteotomy size (p = 0.65), postoperative granuloma (p = 0.89), ICO grading (p = 0.94) or adjuvant received (p = 0.75); while age (p = 0.01) and ostium shape grading (p = 0.04) showed significance. In multivariate analyses, better (deep and wide-open) ostium shape was associated with functional success (p = 0.003) (OR = 2.16,95% CI: 1.10–4.22) while older age with functional failure (p = 0.01) (OR = 0.93,95% CI: 0.88–0.96). ICO grading (p = 0.58) or gender (p = 0.47) did not show statistical significance.

For secondary outcomes, better ostium shape was associated with anatomical success significant in univariate (p = 0.004) but not multivariate analysis (p = 0.10). Grade 4 (deep and wide-open) ostium was associated with functional success in univariate (p = 0.04) and multivariate analysis (p = 0.025) (OR = 2.16,95% CI: 1.10–4.21) (Table 4).

**Table 4:** Secondary outcome — Association of ostium and ICO grading with baseline characteristics and postoperative anatomical & functional outcomes.

	1	2	3	4	p value
**Ostium grade**					
Number of patients	3	28	26	50	n.a.
Group 1:2:3:4 (%)	3:0:0:0 (100:0:0:0)	8:10:6:4 (32:35:24:14)	8:6:7:5 (32:21:28:18)	6:13:12:19 (24:45:48:70)	0.003*
Female (%)	3 (100)	25 (89)	21 (80)	41 (82)	0.30
Right (%)	1 (33)	17 (61)	11 (42)	23 (46)	0.49
Surgeon A (%)	1 (33)	9 (32)	15 (58)	19 (38)	0.50
Granuloma (%)	3 (100)	14 (58)	11 (48)	37 (84)	0.09
Local anesthesia (%)	3:0 (100)	22 (79)	17 (66)	24 (48)	0.001*
Age (years)	61 ± 11.53	61 ± 11.70	66 ± 11.96	59 ± 10.44	0.51
Osteotomy size (mm)	20 ± 5.66	20 ± 2.41	20 ± 3.60	21 ± 3.49	0.05
Anatomical Success (%)	2 (66.7)	24 (86)	23 (89)	50 (100)	0.004*
Functional Success (%)	2 (100)	18 (75)	17 (74)	48 (96)	0.04*
**ICO grade**					
Number of patients	4	11	26	66	n.a.
Group 1:2:3:4 (%)	3:1:0:0 (75:25:0:0)	4:3:3:1 (37:27:27:9)	3:7:7:9 (12:27:27:34)	15:18:15:18 (23:27:23: 27)	0.092
Female (%)	4 (100)	9 (82)	21 (81)	56 (85)	0.87
Right-side (%)	2 (50)	7 (64)	11 (43)	32 (49)	0.72
Surgeon A (%)	1 (25)	3 (27)	10 (39)	30 (46)	0.20
Granuloma (%)	3 (100)	5 (46)	16 (70)	41 (28)	0.59
Local anesthesia (%)	2 (50)	6 (55)	15 (58)	43 (65)	0.34
Age (years)	61 ± 11.53	61 ± 11.70	66 ± 11.96	59 ± 10.44	0.93
Osteotomy size (mm)	20 ± 5.66	20 ± 2.41	20 ± 3.60	21 ± 3.49	0.67
Anatomical Success (%)	2 (50)	8 (73)	25 (96)	64 (97)	<0.001*
Functional Success (%)	1 (50)	7 (88	23 (92)	54 (85)	0.94

Cases with grade 1 ostium shape or ICO were not analyzed due to the limited number. There was no difference of ICO grading among four groups (p = 0.09). Better ICO grading was associated with anatomical success, significant in univariate (p < 0.001) and multivariate analysis (p = 0.03). There was no difference in functional success among different ICO grading (p = 0.94). Cases in group 1, 2 and 3 had similar rate (63.6%, 61.0% and 65.2%) while those in group 4 (bicanalicular stenting) had a significantly higher rate of postoperative granuloma (85.7%, p = 0.04). No difference was found in time to failure or to developing granuloma among the four groups (all p > 0.3). One patient had persistent nasal discharge which resolved after an additional course of oral antibiotic. Another patient had silicone stent dislodged at week 2. These two patients achieved both anatomical and functional success.

## Discussion

Postoperative nasal packing applies pressure for hemostasis, fills surgically created space, forms barrier to developing adhesions and creates moist environment by occlusion to facilitate healing. This is particularly beneficial after MMED with extensive bony and mucosal openings^13^. Absorbable packings include porcine gelatin, oxidized regenerated cellulose or polyethylene glycol with increasing costs^13^. Absorbable gelatin sponge was chosen for its widespread availability and low cost. It is water insoluble and absorbed by phagocytosis in 4–6 weeks^14^. Absorbable gelatin sponge was shown to reduce postoperative bleeding after sinus surgeries. While some studies showed no difference in outcome^13^, others found predisposition to granuloma formation after absorbable gelatin sponge^15^. Non-absorbable packings include expandable polyvinyl acetate and ribbon gauze. The latter conforms and tamponades better to the ostium, offers stronger mucosal compression and allows gentle debridement upon removal^16^. No case of toxic shock syndrome or posterior pack dislocation was noted for the past fifteen years in our institute using non-absorbable packing after endoscopic DCR while patient was put on post-operative antibiotics. Ostium packing promotes healing as an occlusive dressing while pressure *in-situ* decreases mucosal swelling, tamponades the marsupialized lacrimal sac, and maintains edge-to-edge alignment with nasal mucosa which are not sutured after standard endoscopic DCR^14^. One of the main disadvantages of non-absorbable packing is a higher chance of re-bleeding and discomfort during removal^14^. We moistened the ribbon-gauze packing by lacrimal irrigation first right before removal to reduce mucosal microtrauma, bleeding and discomfort.

Bicanalicular intubation was thought to mechanically stent the ICO during early postoperative period when organizing fibrinous materials may clog them. In a previous randomized clinical trial, we found no statistically significant difference whether intubation was used in EEM-DCR for PANDO at the 12-month follow-up^6^. Other studies including systematic review^17^ and meta-analysis^18^ also showed no benefit of stenting in external or endoscopic DCR for PANDO. Some prospective studies and two large, randomized controlled trials^19^ showed benefit of intubation in DCR. A recent meta-analysis showed that the success rate with silicone intubation was significantly better but only powered for the EXT-DCR subgroup while its role in END-DCR remained unanswered. Bicanalicular intubation adds on time, costs, complications and follow-up regime. Stenting may cause inadvertent canalicular injury or even false passaging, promote granuloma formation and punctal erosion. The authors thus advocate stenting only for canalicular obstruction. In this study, patients receiving 8-week bicanalicular stenting achieved similar rate of anatomical success (96.4%) compared to those receiving 1-week packing by ribbon gauze (96.6%) or absorbable gelatin sponge (96%). This was consistent with the results in our previous RCT (SEND) when ostium packing by ribbon gauze only (95.3%) or both stenting and packing (96.3%) were used. Not surprisingly, patients receiving bicanalicular stenting had a higher chance of developing granuloma (87%) compared to other groups (63.4%, p = 0.04).

The shape of DCR ostium depends on the size, location and the characteristics of soft tissue and bony openings. Recent studies showed correlation of ostium shape and functional outcome after endoscopic DCR^20^. Similarly, we found that better-shaped (deep and wide-open) ostium was significantly associated with functional success (odds ratio 2.16, p = 0.03) in multivariate analysis. Patients with wide-open ostia (grade 4: deep with clear border between lacrimal sac and nasal mucosa) showed the best functional outcome which was like the “ice-scoop” type described by Shin *et al*.^21^. We concur that the final ostium shape varies with the underlying size, thickness and mobility of lacrimal sac and the wound healing process with the surrounding nasal mucosa. Previous manometric study showed that lacrimal pump remained functional after DCR^22^. Other retrospective studies also showed that small or fibrotic lacrimal sacs^23^ had a higher chance of functional failure. We postulated that postoperative ostia with better (deep and wide-open) shape were originated from larger, thinner, and more mobile lacrimal sacs while younger patients tend to have healthier, less fibrotic sacs with better “pump function”. Further studies will be required to demonstrate if preoperative lacrimal sac characteristics are more relevant than mechanical adjuvants in determining the functional outcome after MMED for PANDO. Large but fibrotic or atonic sacs were infrequently encountered compared to small and scarred sacs in our practice. The ICO was evaluated by endoscopic visualization of its opening, movement or by dye test^24^. We found that a higher grade ICO was associated with a better anatomical patency in both univariate and multivariate analyses. Since ICO is where canaliculi open into the lacrimal sac, higher grades (less obstructed ICO) implies better anatomical patency.

We acknowledged the retrospective, non-randomized nature of our study involving only 103 patients. After the SEND study^6^, which we showed no difference in 12-month outcome between stenting and packing or packing alone, there was no institutional protocol as to whether any mechanical adjuvant including stenting and/or packing be used routinely in PANDO. While preoperative and intraoperative variables were comparable, groups were consisted of different number of patients due to incomplete follow-up. The small number of patients within subgroups may also limit meaningful analyses. We did notice that 12-month success rate were similar between Group 2 (pack without stent) in the current study (SPEND) versus Group 2 (pack without stent) in SEND.

## Conclusions

Firstly, 1-week ostium packing was found to be as effective a mechanical adjuvant as 8-week bicanalicular intubation in improving anatomical outcome after MMED for PANDO. Secondly, mechanical adjuvants were found useful only to improve anatomical but not functional success. Thirdly, non-surgical factors including patient’s age and ostium shape, the latter reflects the underlying lacrimal sac and ostium healing process, were associated with functional outcome, which is a more important endpoint than anatomical patency. To our knowledge, this is the first study comparing stenting and different packing (non-absorbable and absorbable) in MMED for PANLDO. Prospective randomized trials will help to address the specific role(s) of surgical adjuncts (either mechanical and/or pharmacological e.g. mitomycin) to improve functional outcomes of MMED, especially in high-risk cases e.g. advanced age or small and scarred lacrimal sac.

## Data Availability

The datasets generated during and/or analysed during the current study are available from the corresponding author on reasonable request.
